# Impact of Ankle Injury History and Pre-National Football League Draft Training on Lower Limb Coordination During Running: A Pilot Study

**DOI:** 10.7759/cureus.93887

**Published:** 2025-10-05

**Authors:** James Ro, Ashlyn E Brushwood, Monique Mokha

**Affiliations:** 1 Medicine, Nova Southeastern University Dr. Kiran C. Patel College of Allopathic Medicine, Fort Lauderdale, USA; 2 Health and Human Performance, Nova Southeastern University Dr. Kiran C. Patel College of Osteopathic Medicine, Fort Lauderdale, USA

**Keywords:** american football, ankle sprain, coordination variability, intersegmental coordination, running biomechanics

## Abstract

Objective

Ankle sprains are one of the most common injuries sustained by American football athletes. Players with a recent history of ankle sprain, even when asymptomatic, may have sensorimotor impairments that affect control of the hip and knee during dynamic activities such as speed running. Among players preparing for the National Football League (NFL) draft, these functional deficits have wide-ranging effects and warrant systematic investigation. This study aimed to determine the influence of a recent history of ankle sprain (≤6 months) and a six-week NFL draft preparation training camp on hip-ankle (HA) and knee-ankle (KA) coordination. Specifically, we examined HA and KA coordination angles and variability using vector coding. We hypothesized that the group with a recent ankle sprain history (RASH) would express less ankle-dominant patterns, and these would improve post-training, and the RASH group would show impaired neuromuscular adaptability as evidenced by lower coordination variability, but this would improve post-training, through increased variability.

Materials and methods

This study analyzed a sample of 12 athletes with a recent ankle sprain history (RASH) and compared their running mechanics to 12 matched controls with no recent ankle sprain history (non-RASH). Running mechanics during a five-second 23 km/h run were evaluated pre- and post-training using a 10-camera motion capture system. Coordination was quantified into angles using vector coding, and coordination variability was quantified using circular statistics, with variability expressed as the standard deviation derived from the mean resultant length of angular data. Data for both were derived over four subphases of the running gait cycle: first half stance, second half stance, first half swing, and second half swing. A 2 (RASH and non-RASH) × 2 (pre and post) ANOVA were used to assess the influence of ankle sprain history and training on HA and KA coordination and coordination variability (p ≤ 0.05).

Results

No significant group × time interactions or main effects were found in any of the subphases for coordination angles or variability for HA and KA (p > 0.05). For HA, there were medium effect sizes in coordination angle for the second half of stance (η²ₚ = 0.075) and coordination variability for the second half of swing (η²ₚ = 0.121). The RASH group had larger increases in HA angle from training (212.7 ± 102.7° (pre) versus 235.5 ± 91.2° (post)) than the non-RASH group (268.7 ± 56.8° (pre) versus 265.5 ± 48.2° (post)). Further, the RASH group shifted from an in-phase hip-dominant to an in-phase ankle-dominant pattern in the second half of stance. For KA, there were medium effect sizes for the first half stance (η²ₚ = 0.093) and second half swing (η²ₚ = 0.091). Specifically, for the first half stance, the RASH group changed from anti-phase ankle to in-phase ankle control (97.0 ± 19.5° (pre) versus 85.5 ± 29.4° (post)), while the non-RASH group was stable at an anti-phase ankle pattern (93.6 ± 19.8° (pre) versus 95.6 ± 19.8° (post)). For the second half swing, both groups produced in-phase knee-dominant patterns, but the RASH group decreased their coordination angle by 4.4°, and the non-RASH group increased their coordination angle by 3.8°.

Conclusions

Highly skilled athletes with a recent history of ankle sprains adopt compensatory joint coupling strategies that persist even in the absence of symptoms and can be modified through training.

## Introduction

Ankle sprains are among the most common injuries in college athletes, accounting for approximately 7.3%-15% of all injuries across sports [1-3]. In American football, ankle sprains rank second only to concussions in frequency [4]. Among National Football League (NFL) draft preparation participants, 53% report a history of ankle injuries [5], with particularly high rates observed in running backs (61.9%), offensive linemen (60.3%), and tight ends (59.4%). Kickers/punters (23.3%) and long snappers (37.5%) had the lowest injury rates [5]. Further, the recurrence of ankle sprains is high [6,7], and repeated episodes often result in residual sensorimotor deficits [8].

Biomechanically, athletes with a history of ankle sprains often demonstrate altered lower limb gait kinematics. These include limited dorsiflexion range of motion (ROM) in the stance phase [9], limited and larger variability in dorsiflexion across running speeds [10], and compensatory changes at the hip and knee [11,12]. For example, during dynamic activities, individuals with chronic ankle instability may exhibit delayed knee flexion or increased hip adduction, potentially redistributing load and impacting intersegmental coordination.

Intersegmental coordination is the timing and interaction between adjacent joints. The study of intersegmental coordination has clinical application. It plays a critical role in efficient gait and injury resilience [13]. Coordination variability reflects the motor control system’s ability to adapt to environmental or task constraints [14]. While healthy variability promotes flexible motor control, excessive or insufficient variability may signal dysfunctional movement. Reduced coordination variability has been linked to joint stiffness and limited movement options post-injury [15], while elevated variability may indicate instability and risk for reinjury, as seen in anterior cruciate ligament-deficient knees [16] or shoulder mechanics in swimmers and overhead athletes [17]. In athletes with a history of ankle sprain, studies suggest a narrower range of coordination patterns and a less adaptable control strategy during walking and running [12,18]. This rigidity could elevate the risk of subsequent injuries, particularly in sports such as American football, which require rapid acceleration and deceleration, changes in direction, and high force absorption. Participants in the current study were NFL draft-eligible players. At their tryout, they will undergo a comprehensive medical examination that includes a review of their medical history, a body composition assessment, a psychological evaluation, on-field position drills, and physical skills testing. The latter is made up of a 36.6-m sprint with split times reported at 9.1 and 18.3 m, vertical and horizontal jump distances, an 18.3-m shuttle and three-cone drill times, and a 102.1-kg bench press for maximum repetitions. Thus, optimizing lower extremity function is critical to prevent injuries and allow for peak performance at their tryout.

Vector coding is a nonlinear technique from the dynamical systems framework that quantifies joint coordination by analyzing the relative motion between two segments over time [19]. Modified vector coding (MVC) has been applied to identify altered hip-ankle (HA) or knee-ankle (KA) coupling in individuals with ankle sprain history (specifically, chronic ankle instability) and to distinguish between patients with and without residual symptoms [20]. When used in conjunction with angle-angle plots, MVC enables precise insights into motor coordination patterns and provides a valuable tool for evaluating recovery and retraining effectiveness in post-injury rehabilitation [21] and other training interventions.

Despite the known prevalence of ankle injuries in elite football players, no studies to date have examined how elite-level training affects intersegmental coordination in athletes with and without ankle sprain history. It remains unclear whether high-level neuromuscular training can restore joint coupling patterns and coordination variability in those with impaired motor flexibility. Therefore, the purpose of this study was to compare elite American football players with and without recent ankle sprain history (RASH) and how they coordinate movement across the hip, knee, and ankle during high-speed running. Additionally, we sought to determine if an NFL draft preparation training camp could significantly change intersegmental coordination angles and variability. Using modified vector coding, we analyzed hip-ankle and knee-ankle coordination and coordination variability before and after a six-week NFL draft preparation training program. We hypothesized that the RASH group would express less ankle-dominant patterns, especially in the late stance phase, and the patterns would improve to more ankle-dominant post-training, and the RASH group would demonstrate reduced coordination variability at the pre-test, reflecting motor control rigidity, but would show increased variability post-training, indicating improved neuromuscular adaptability.

## Materials and methods

Experimental approach

This study was part of a larger study monitoring pre-post changes in body composition, and vertical jump and speed running mechanics throughout a six-week training camp designed to prepare eligible American football players for the 2025 NFL draft. The study procedures were conducted at the start and end of the training camp. This study was approved by the university’s Institutional Review Board (#2018-684) and complied with the Declaration of Helsinki [22]. All subjects provided written informed consent.

Subjects

A sample of convenience of 67 adult American football players was recruited from a group of players enrolled in a specialized training camp at a local performance center for the 2025 NFL draft (six times per week). All completed the testing. Twelve of 67 players tested (18%) (age: 23.0 ± 1.1 years, height: 1.85 ± 0.6 m, mass: 102.1 ± 18.5 kg) presented with a recent ankle sprain history (RASH) within the six months of the start of training camp and were the experimental group in this study. A control group consisted of 12 healthy players without a history of lower extremity injury in the preceding six months (age: 22.8 ± 0.9 years, height: 1.88 ± 0.09 m, mass: 105.5 ± 20.3 kg) matched by player position category and treadmill run speed. Player position categories were Big (e.g., linemen), Big-skill (e.g., tight ends and linebackers), and Skill (e.g., wide receivers and defensive backs), and run speeds were between 21.6 and 23.4 km/h. All subjects were cleared for participation by board-certified and licensed medical practitioners.

Protocol

Subjects reported to the Sport Performance and Gait Laboratory in groups of three at a designated time over 2 ½ days at both the start and end of the six-week training camp. The training camp was divided into three microcycles that included drill specificity training, player position specificity training, resistance training, and recovery-based movement (e.g., pool and soft tissue massage). Table 1 and Table 2 present descriptions of the training used by the coaches. A sample of a complete training camp showing the specific exercises, volumes, and intensities can be found at https://simplifaster.com/articles/tracking-training-athletes-nfl-combine/. While these exercises and drills do not target the ankle joint complex, they employ it in a multi-planar and multi-joint manner.

**Table 1:** Description of the training volumes to raise the force threshold over the three microcycles

Microcycle	Resisted work (heavy)	Resisted work (light)	Plyometrics	Technical drills
1 (weeks 1-2)	Very high volume	Low volume	Low volume	Medium volume
2 (weeks 3-4)	Medium volume	Low volume	Medium volume	High volume
3 (weeks 5-6)	Low volume	Very high volume	Very high volume	Low volume

**Table 2:** Description of the training volumes to raise the threshold to apply force over the three microcycles

Microcycle	Free sprints (short)	Free sprints (long)	Assisted sprints (short)	Assisted sprints (long)
1 (weeks 1-2)	High volume	Low volume	Low volume	N/A
2 (weeks 3-4)	Medium volume	Low volume	Medium volume	High volume
3 (weeks 5-6)	Low volume	High volume	High volume	High volume

During data collection, subjects wore compression shorts and their own running shoes. After explaining the study and procuring written informed consent, anthropometric measures were obtained according to the specifications of the Vicon Nexus Plug-in Gait lower body model (Vicon, Centennial, CO). To assure accuracy, the researchers used a felt-tip marker to identify the anterior superior iliac spines since these landmarks are used for pelvis width, leg length, and marker placement. Following anthropometrics, subjects completed a standardized 25-minute warm-up led by a single coach consisting of dynamic stretching, muscle readiness, and reactivity exercises. Then, sixteen 14 mm retroreflective markers were placed bilaterally on the participant’s anterior superior iliac spines, lateral knee joints, lateral ankle malleoli, lateral thighs, lateral lower legs, heads of the second metatarsals, calcanei, and posterior superior iliac spines according to the specifications of Vicon’s Plug-in Gait lower body model. Then, a local calibration was performed. The running trial took place on an instrumented split-belt treadmill (Bertec Corporation, Columbus, OH), with subjects running on one side at zero incline. Subjects began by walking on the treadmill at 3.6 km/h for 2-3 minutes. Speed was increased 3.6 km/h in one-second increments, with three-second pauses at jogging (~12.6 km/h) and fast jogging (~18.0 m/s) speeds to facilitate comfort. When the player reached preferred maximum speed (the maximum speed allowed by the treadmill was 23.4 km/h), they ran for 1-2 seconds before a recording was taken. Capture time was five seconds, selected to mimic most 36.6-m (40-yd) run durations at the NFL draft preparation, and it allowed for at least eight strides. Hafer and Boyer reported that eight running strides are necessary to calculate coordination variability sufficiently [23]. At the completion of the trial, subjects transferred their weight to the non-moving belt, and the running treadmill belt was decelerated to a stop. The mean running speed was 23 km/h, and the speed achieved at the pre-test was replicated at the post-test. Only one running trial was collected at both testing sessions. Subjects were instructed to wear the same shoes to the post-test. However, the researchers did not ensure compliance. No subjects wore ankle joint prophylactic braces or tape.

Instrumentation

Kinematic and kinetic data were captured in a laboratory using a 10-camera 3D motion analysis system (Vicon, Centennial, CO), sampled at 120 Hz, and recorded synchronously with an instrumented split-belt treadmill (Bertec Corporation, Columbus, OH), which collected data at 1000 Hz. The motion analysis system was calibrated on the day of each data collection session to an accuracy defined by a 3D residual below 0.20 mm. Lower limb length, pelvis and lower limb breadth, and total body mass were measured using a standard fiberglass tape measure, anthropometers (Lafayette Instruments, Lafayette, IN), and an InBody 270 multifrequency bioelectrical impedance device (InBody USA, Cerritos, CA), respectively. Height was self-reported.

Data processing

**Figure 1:**
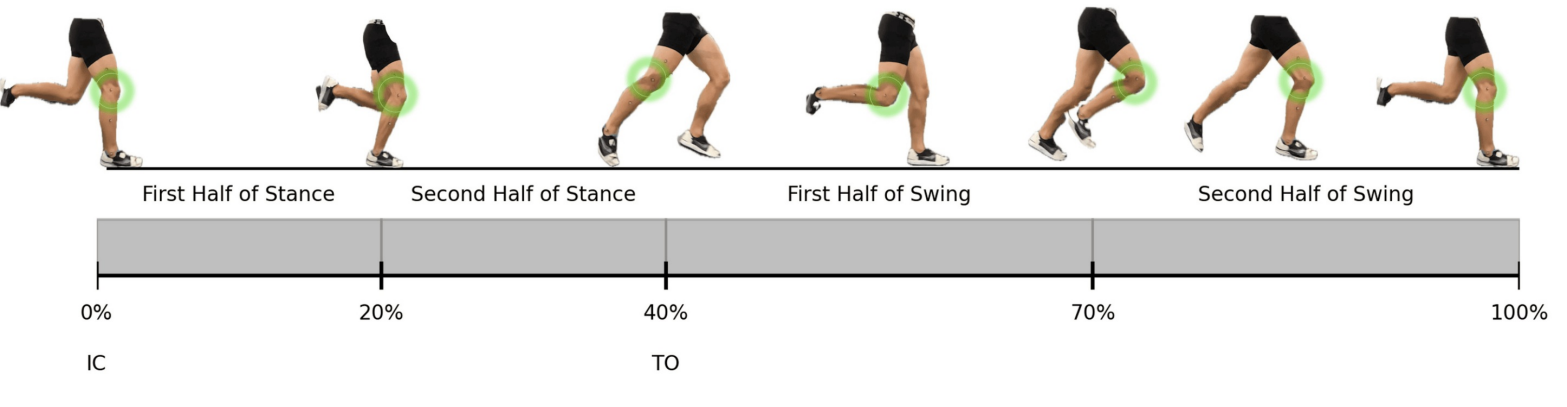
Phases of the running gait cycle This diagram shows the four subphases of the gait cycle for a single limb. IC: initial contact, TO: toe off

The outcome variables were the coordination angles of the hip-ankle (HA) and knee-ankle (KA), as well as coordination variability in the sagittal plane. Marker coordinates were labeled and gap-filled, and the angles of the hip, knee, and ankle joints were calculated using Vicon Nexus software (version 2.15). As subjects ran on one side of the split-belt treadmill, gait cycles were visually inspected to identify left and right ground contact and foot-off events in the software, based on vertical ground reaction forces. Marker trajectories and ground reaction forces were filtered using a third-order low-pass Butterworth filter with cutoff frequencies of 10 Hz [24] and 50 Hz (to ensure accurate detection of high-impact activity temporal events), respectively. Gaps in marker trajectories due to occlusion or dropout were filled using a quintic spline interpolation. No gaps were greater than 10 frames, and visual inspection of the X, Y, and Z residuals (raw, filtered) showed no outliers. The data were then post-processed in a custom MATLAB® program (MathWorks, Natick, MA) that included the biomechZoo toolbox [25], where the stance phase was defined as the period of foot-ground contact with the treadmill when the vertical ground reaction force exceeded 20 N. The running gait cycle for the limb was interpolated at 101 points and divided into four phases for more specific analysis: first half stance (0%-20%), second half stance (21%-40%), first half swing (41%-70%), and second half swing (71%-100%) (Figure 1) [26].

**Figure 2:**
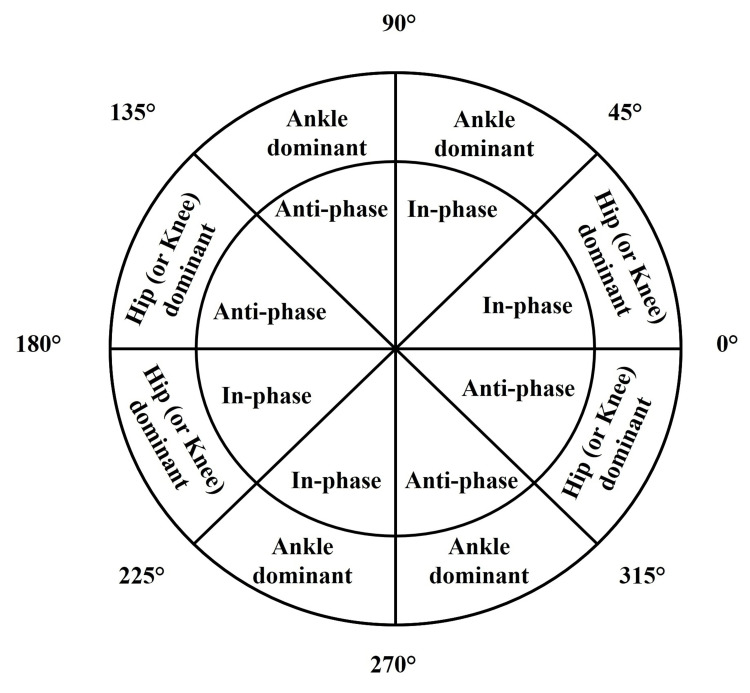
Polar plot showing the coordination pattern classification This polar plot was adapted from Needham et al. [21] and Needham et al. [28], and provides the coordination pattern classification between two joints as either in-phase or anti-phase and based on joint dominancy.

Coordination angles between the hip and ankle, and the knee and ankle joints were derived using a modified vector coding approach based on the mean joint angle trajectories across all strides in the five-second trial. Angle-angle plots with the ankle position at the X-axis and the proximal joint (hip or knee) position at the Y-axis. Coordination angles represent the vector orientation between two adjacent time points on an angle-angle plot relative to the right horizontal [27]. Coordination angles were calculated from the first derivatives of joint angles (e.g., ankle versus hip), representing the relative phase of motion throughout the normalized running gait cycle. Circular means and standard deviations were computed across the four subphases to characterize average coordination patterns and their within-cycle variability. The following formula was used to quantify the orientation of the vector of the coupling angle:

$&theta;_{coord}(t) = mod(tan⁻&sup1;(∆&theta;_{prox}(t) / ∆&theta;_{dist}(t)), 360&deg;$

prox(t) is the change in the proximal joint angle (e.g., hip or knee) at time t, and dist(t) is the change in the distal joint angle (e.g., ankle) at time t. Angles were wrapped to the range of 0-360° using the modulo operation in MATLAB® to represent continuous angular coordination. Figure 2 depicts the coordination pattern that a vector angle indicates when it falls onto each of the quadrants of a polar plot (adapted from Needham et al. [21] and Needham et al. [28]). The specific coordination patterns are in-phase proximal (hip or knee) dominancy (0-45° or 180-225°), in-phase ankle dominancy (45-90° or 225-270°), anti-phase ankle dominancy (90-135° or 270-315°), and anti-phase proximal (hip or knee) dominancy (135-180° or 315-360°). In-phase denotes that both joints rotate in the same direction, whereas anti-phase means the joints rotate in opposite directions. The dominant joint is the one that undergoes the greater angular rotation change in each instant of time. Further, vector angles of 90° and 270° indicate that one joint in the couple is moving while the other is stationary. Circular means and circular standard deviations were calculated using the Circular Statistics Toolbox in MATLAB® to quantify average joint coordination angles and their variability.

For each subphase, the variability of coordination angles across strides was computed using the standard deviation: 

$\text{var}_{\text{coord}} = \sqrt{-2 \ln(R)} \cdot \left( \frac{180^\circ}{\pi} \right)$

$R = \frac{1}{n} \sum_{i=1}^{n} e^{j\theta_i}$ is the mean resultant length of the circular data, and $\theta_{i}$ are the coordination angles in radians for stride i. Larger angles indicated increased variability.

Statistical analysis

Group demographic information was compared using independent t-tests in the Statistical Package for Social Sciences (SPSS) version 29.0 (SPSS, Inc., Chicago). Multiple 2 × 2 (group × time) analysis of variance (ANOVA) were used to evaluate the influence of RASH and training (pre and post) on HA and KA coordination angles and variability across four subphases of the running gait cycle. The level of significance for this exploratory study was set a priori as p < 0.05. Effect sizes were determined using partial eta-squared (η²ₚ) thresholds of 0.01, 0.06, and 0.14 interpreted as small, medium, and large, respectively [29]. Ninety-five percent confidence intervals (CI) for η²ₚ were calculated within SPSS using its R integration plug-in, based on the noncentral F distribution method [30].

## Results

Independent t-tests showed no significant differences in age (p = 0.698), mass (p = 0.677), or height (p = 0.386) between the recent ankle history (RASH) group and the control, non-recent ankle sprain history (non-RASH) group.

Table 3 and Table 4 depict means and standard deviations of coordination angles and coordination variability, respectively, for both groups before and after training camp.

**Table 3:** Coordination angle means and standard deviations in degrees for RASH and non-RASH groups RASH: recent ankle sprain history, non-RASH: non-recent ankle sprain history

	RASH		Non-RASH	
	Pre	Post	Pre	Post
Hip-ankle				
1st half stance	153.9 ± 10.8	154.1 ±10.7	153.6 ± 6.5	152.4 ± 6.9
2nd half stance	212.7 ± 102.7	235.5 ± 91.2	268.7 ± 56.8	265.5 ± 48.2
1st half swing	16.9 ± 5.1	19.1 ± 6.7	18.6 ± 9.3	20.1 ± 8.5
2nd half swing	184.1 ± 37.4	187.3 ± 36.9	174.9 ± 31.1	184.1 ± 30.2
Knee-ankle				
1st half stance	97.0 ± 19.5	85.5 ± 29.4	93.6 ± 19.8	95.6 ± 19.8
2nd half stance	321.5 ± 21.2	317.3 ± 23.1	317.9 ± 15.4	315.9 ± 17.5
1st half swing	34.4 ± 8.2	34.0 ± 10.1	31.1 ± 8.7	32.8 ± 7.7
2nd half swing	188.6 ± 7.5	184.2 ± 12.2	186.2 ± 5.4	186.0 ± 5.7

**Table 4:** Coordination variability means and standard deviations in degrees for RASH and non-RASH groups RASH: recent ankle sprain history, non-RASH: non-recent ankle sprain history

	RASH		Non-RASH	
	Pre	Post	Pre	Post
Hip-ankle				
1st half stance	44.2 ± 5.6	45.7 ± 4.2	42.7 ± 5.0	43.7 ± 3.9
2nd half stance	64.3 ± 8.1	63.8 ± 9.7	67.1 ± 6.1	65.7 ± 8.7
1st half swing	14.6 ± 7.6	15.7 ± 5.1	15.4 ± 6.7	17.0 ± 6.3
2nd half swing	60.7 ± 8.1	59.5 ± 7.6	62.0 ± 7.6	63.2 ± 7.3
knee-ankle				
1st half stance	69.1 ± 6.7	69.2 ± 6.1	68.5 ± 4.4	68.6 ± 2.7
2nd half stance	49.0 ± 7.5	48.9 ± 4.9	50.3 ± 5.0	50.7 ± 4.1
1st half swing	55.8 ± 6.0	53.6 ± 8.1	53.1 ± 7.0	52.8 ± 7.7
2nd half swing	45.5 ± 11.5	46.4 ± 15.3	37.3 ± 10.0	38.6 ± 9.9

**Figure 3:**
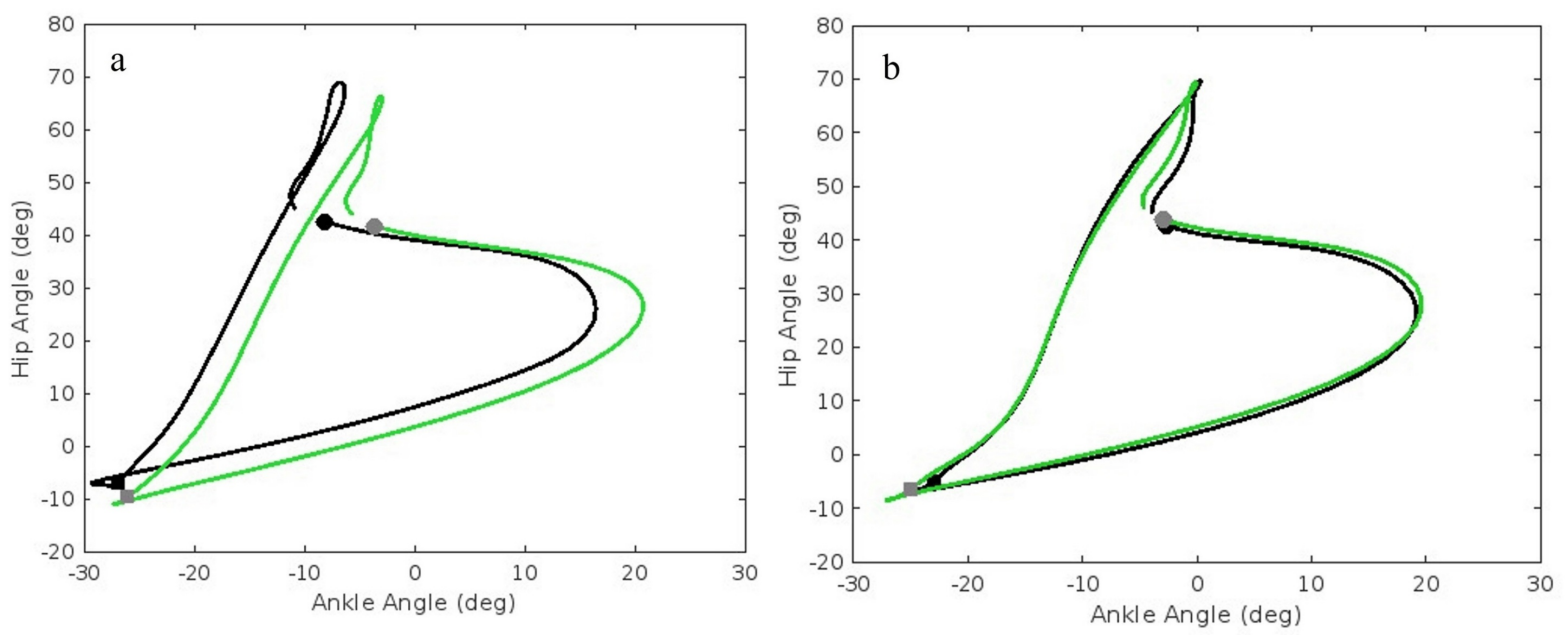
Hip-ankle angle-angle plots for (a) RASH and (b) non-RASH ● indicates initial contact; ■ indicates take-off. The angle-angle plots visualize the pre (black line) and post (green line) sagittal plane hip motion relative to ankle motion during the running gait cycle, averaged over all steps. RASH: recent ankle sprain history, non-RASH: non-recent ankle sprain history

**Figure 4:**
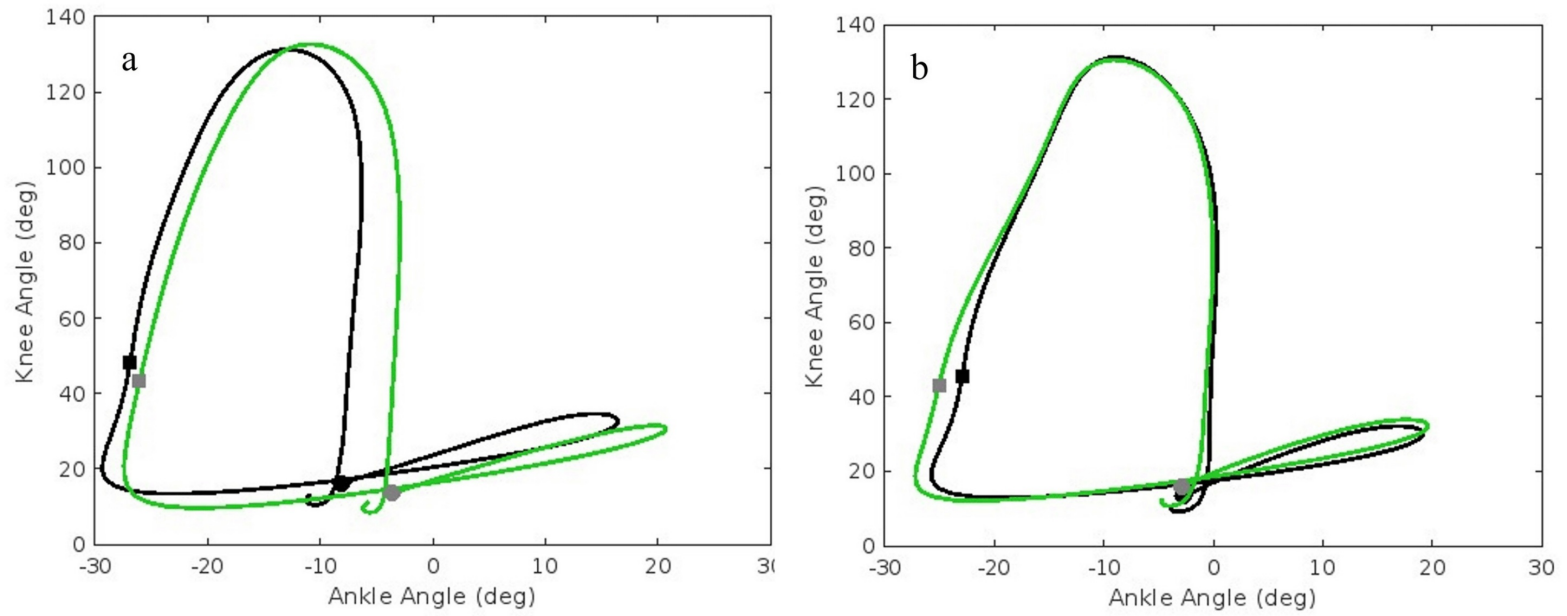
Knee-ankle angle-angle plots for (a) RASH and (b) non-RASH ● indicates initial contact; ■ indicates take-off. The angle-angle plots visualize the pre (black line) and post (green line) sagittal plane knee motion relative to ankle motion during the running gait cycle, averaged over all steps. RASH: recent ankle sprain history, non-RASH: non-recent ankle sprain history

While both groups exhibited similar baseline HA and KA coordination patterns, the RASH group showed noticeable adaptations from pre- to post-training, as observed in the angle-angle plots (Figure 3 and Figure 4). RASH appears to have adopted a more flexible motor control strategy while becoming less proximally dominant. Specifically, HA coupling for the RASH group shows increased hip extension at take-off and increased ankle dorsiflexion at midstance. For KA coupling, notable adaptations included greater ankle dorsiflexion during the swing phase, accompanied by increased swing phase knee flexion and reduced ankle plantarflexion in the second half of stance.

The plots were generated using MATLAB®, and the source code is available at https://github.com/mmokha/joint_coord.git for reproducibility.

A series of 2 × 2 (group × time) mixed model ANOVAs were conducted to evaluate the effects of recent ankle injury history and training on HA and KA coordination angles and variability across four subphases of the running gait cycle.

**Figure 5:**
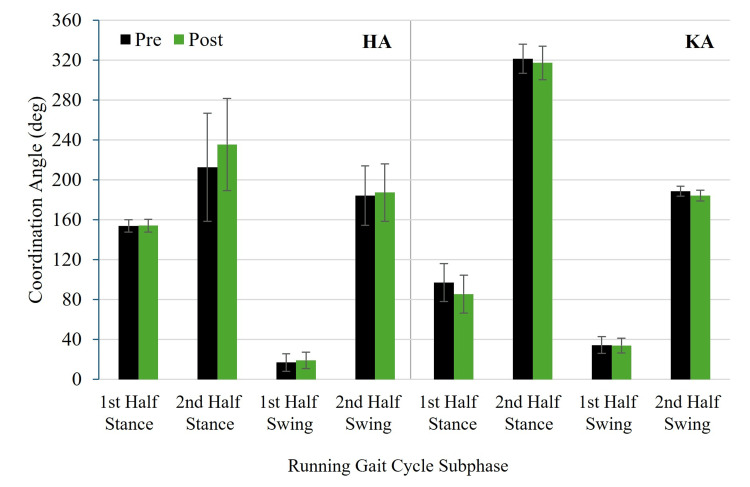
Mean pre- and post-test HA and KA coordination angles for RASH at each subphase of the running gait cycle HA: hip-ankle, KA: knee-ankle, RASH: recent ankle sprain history

**Figure 6:**
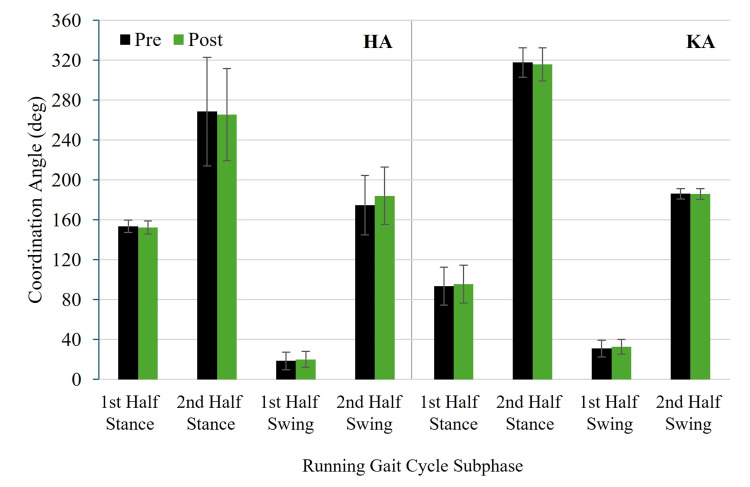
Mean pre- and post-test HA and KA coordination angles for non-RASH at each subphase of the running gait cycle HA: hip-ankle, KA: knee-ankle, non-RASH: non-recent ankle sprain history

**Figure 7:**
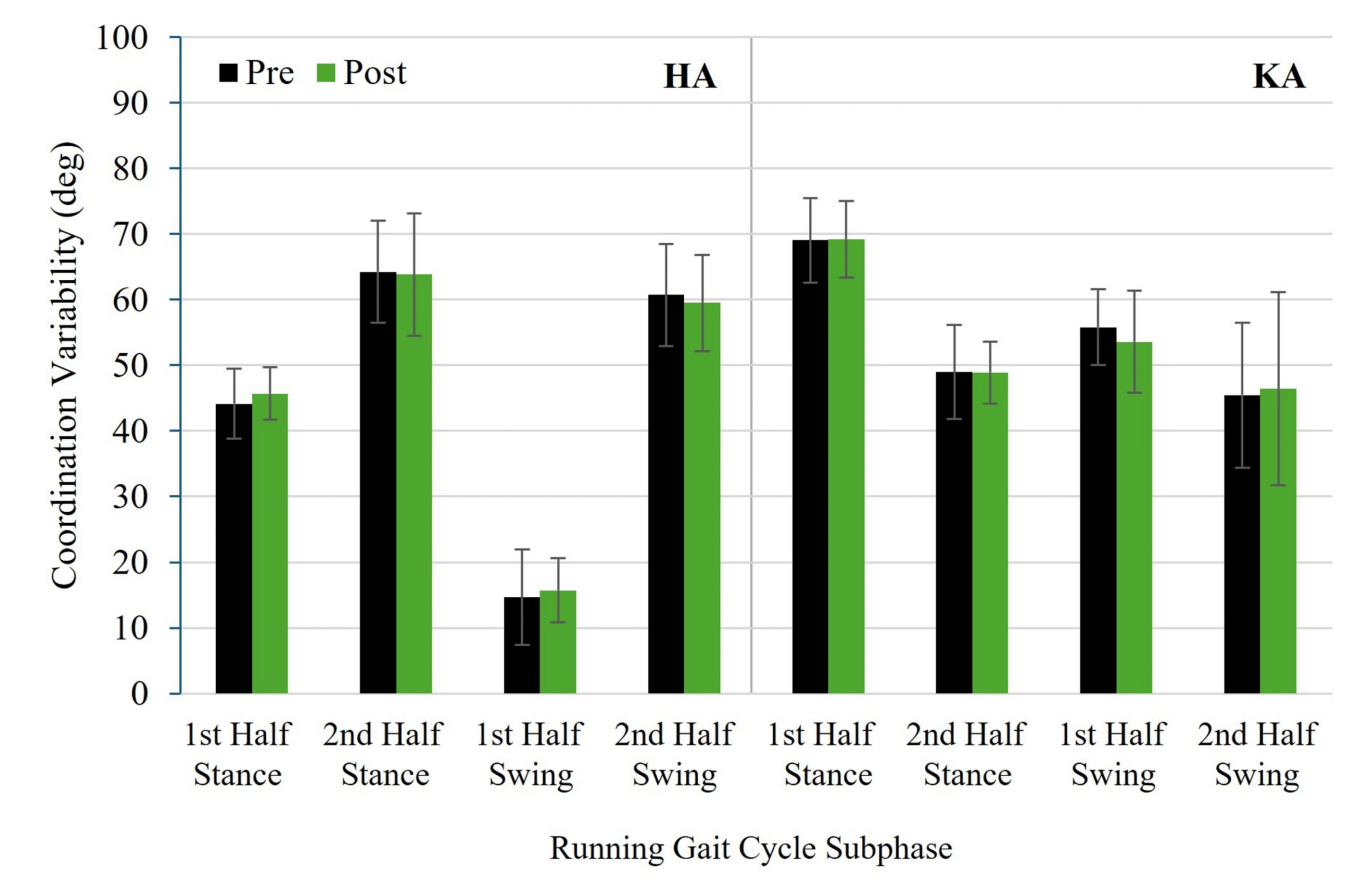
Mean pre- and post-test HA and KA coordination variability for RASH at each subphase of the running gait cycle HA: hip-ankle, KA: knee-ankle, RASH: recent ankle sprain history

**Figure 8:**
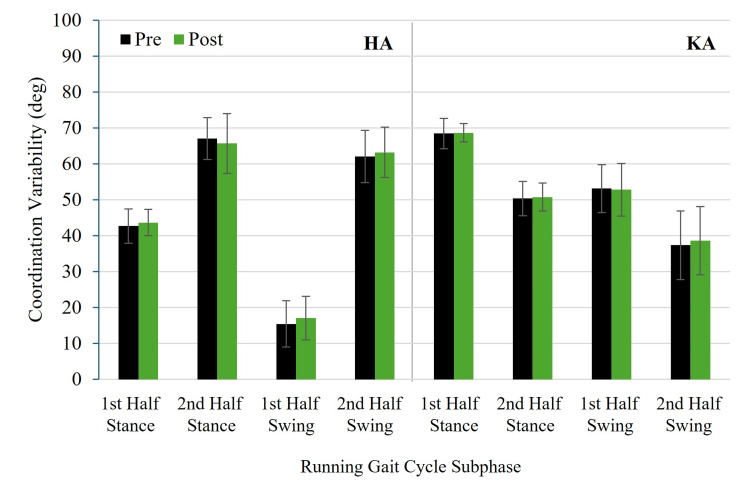
Mean pre- and post-test HA and KA coordination variability for non-RASH at each subphase of the running gait cycle HA: hip-ankle, KA: knee-ankle, non-RASH: non-recent ankle sprain history

Figure 5 and Figure 6 show pre- and post-test HA and KA coordination angles for RASH and non-RASH groups, respectively. Figure 7 and Figure 8 illustrate pre- and post-test HA and KA coordination variability for RASH and non-RASH groups, respectively.

Hip-ankle coordination angles

No significant group × time interactions were observed for any subphase for HA coordination angles: first half stance, F(1,22) = 0.259, p = 0.616, η²ₚ = 0.012, 95% CI: 0.000, 0.213; second half stance, F(1,21) = 1.693, p = 0.207, η²ₚ = 0.075, 95% CI: 0.000, 0.339; first half swing, F(1,22) = 0.082, p = 0.777, η²ₚ = 0.004, 95% CI: 0.000, 0.170; and second half swing, F(1,22) = 0.234, p = 0.633, η²ₚ = 0.011, 95% CI: 0.000, 0.209 (Figure 5 and Figure 6). Although none of these interactions reached statistical significance, the medium effect size for the second half of stance (η²ₚ = 0.075) may reflect a potentially meaningful group × time difference, specifically that training increased the coordination angle of RASH (212.7 ± 102.7° (pre) versus 235.5 ± 91.2° (post)), but not non-RASH (268.7 ± 56.8° (pre) versus 265.5 ± 48.2° (post)) moving RASH from in-phase hip-dominant to in-phase ankle-dominant control. Additionally, the main effects of group and time were not significant in the subphases (p > 0.05).

Knee-ankle coordination angles

No significant group × time interactions were found for any subphase for KA coordination angles: half stance, F(1,22) = 2.257, p = 0.147, η²ₚ = 0.093, 95% CI: 0.000, 0.357; second half stance, F(1,22) = 0.200, p = 0.659, η²ₚ = 0.009, 95% CI: 0.000, 0.210; first half swing, F(1,22) = 0.430, p = 0.519, η²ₚ = 0.019, 95% CI: 0.000, 0.236; or second half swing, F(1,22) = 2.200, p = 0.152, η²ₚ = 0.091, 95% CI: 0.000, 0.354 (Figure 5 and Figure 6). Although not statistically significant, the interaction effects for first half stance (η²ₚ = 0.093) and second half swing (η²ₚ = 0.091) were medium in magnitude, indicating potentially meaningful group × time differences. Specifically for the first half swing, RASH changed from anti-phase ankle to in-phase ankle control (97.0 ± 19.5° (pre) versus 85.5 ± 29.4° (post)), while non-RASH was stable at an anti-phase ankle pattern (93.6 ± 19.8° (pre) versus 95.6 ± 19.8° (post)). For the second half swing, both groups produced in-phase knee-dominant patterns, but RASH decreased the coordination angle by 4.4°, and non-RASH increased the coordination angle by 3.8°. The main effects of group and time were also not significant across the four subphases (p > 0.05).

Hip-ankle coordination variability

No significant group × time interactions were observed for HA coordination variability in first half stance (F(1,22) = 0.802, p = 0.380, η²ₚ = 0.035, 95% CI: 0.000, 0.271), second half stance (F(1,22) = 0.244, p = 0.626, η²ₚ = 0.011, 95% CI: 0.000, 0.218), first half swing (F(1,22) = 3.024, p = 0.096, η²ₚ = 0.121, 95% CI: 0.000, 0.389), or second half swing (F(1,22) = 1.391, p = 0.251, η²ₚ = 0.059, 95% CI: 0.000, 0.312). Although the interaction during the first half swing was not statistically significant, the effect size (η²ₚ = 0.121) was medium, suggesting a potentially meaningful group × time difference. Specifically, non-RASH (15.4 ± 6.7° (pre) versus 17.0 ± 6.3° (post)) increased variability more than RASH (14.6 ± 7.6° (pre) versus 15.7 ± 5.1° (post)). No significant group or time main effects were found (all p > 0.05).

Knee-ankle coordination variability

For KA coordination variability, no significant group × time interactions were found in first half stance (F(1,22) = 0.567, p = 0.459, η²ₚ = 0.025, 95% CI: 0.000, 0.251), second half stance (F(1,22) = 0.021, p = 0.887, η²ₚ = 0.001, 95% CI: 0.000, 0.127), first half swing (F(1,22) = 1.349, p = 0.258, η²ₚ = 0.058, 95% CI: 0.000, 0.309), or second half swing (F(1,22) = 1.166, p = 0.292, η²ₚ = 0.050, 95% CI: 0.00, 0.298). Although the interaction during the first half swing was not statistically significant, the effect size (η²ₚ = 0.058) approached the criteria for a medium effect, suggesting a potentially meaningful group × time difference. RASH (55.8 ± 6.0° (pre) versus 53.6 ± 8.1° (post)) had greater reductions in variability in the first half of swing than non-RASH (53.1 ± 7.0 (pre) versus 52.8 ± 7.7° (post)). No significant group or time main effects were found (all p > 0.05).

Figure 7 and Figure 8 depict pre- and post-test HA and KA coordination variability for RASH and non-RASH, respectively.

## Discussion

The purpose of this pilot study was to compare lower limb sagittal plane coordination angles and coordination variability of elite American football players with and without recent ankle sprain history before and after an NFL draft preparation training camp. To the authors’ knowledge, this is the first study to investigate the effects of training on intersegmental coordination during high-speed running in American football players attempting to make the NFL. The hypothesis that players with a recent history of ankle sprain (≤6 months) would demonstrate reduced coordination variability at the pre-test, reflecting motor control rigidity, and increased variability post-training, indicating improved neuromuscular adaptability, was formally rejected. However, several findings for coordination angles and coordination variability for hip-ankle (HA) and knee-ankle (KA) couplings across the running gait subphases yielded medium to large effect sizes and an overall reduction in proximal dominance. Despite suffering from a recent ankle sprain, subjects may have developed compensatory coordination mechanisms to shift their body weight or utilize different muscles and tissues to reduce stress on the previously injured joint, thereby passively preventing tissue overuse [31]. Given the highly skilled and elite nature of the subject pool, where small changes may be meaningful, these results warrant further attention as they suggest a potential effect of athletic training on motor flexibility restoration.

Hip-ankle coordination

While we were unable to show statistically significant differences between the coordination angles and variability in HA coordination, the angle-angle plot reveals noticeable differences in the running motion within the RASH group following NFL draft preparation training. The RASH group data indicated an increase in hip extension during the takeoff phase of their running gait while also increasing their dorsiflexion of the ankle at the point of contact and throughout the midstance phase. In the first half phases of stance and swing, there is a noticeable increase in coordination variability following the six-week training camp, demonstrating less rigidity and possibly decreased risk of future injury [12,18]. Recent ankle sprain history, even in the absence of symptoms, appears to subtly alter HA coupling. Specifically, there was a pattern of greater hip dominance, which the authors attribute to a compensatory proximal strategy.

First Half Stance

In the first half of stance, both groups exhibit relatively anti-phase, hip-dominant coordination patterns, as evidenced by angles averaging between 152.4° and 154.1° before and after training camp. This makes sense given the role of the ankle during initial contact and loading that occurs in this subphase. Motion is greater from the hip compared to the ankle during this subphase, which reflects the expected biomechanics of high-speed running. The ankle plays a critical stabilizing role from initial contact to midstance, while the hip generates greater angular motion to manage the pelvis and trunk position [26]. Variability subtly increased for both groups, which can be interpreted as a positive training adaptation. However, coordination variability was slightly lower in the non-RASH group, indicating a more consistent running gait, possibly due to reduced demand of neuromuscular adaptation, as these athletes did not have to compensate movement patterns from ankle injury [15]. However, the effect size was small (0.012).

Second Half Stance

A noticeable difference is visualized in the second-half stance phase of the running gait between the RASH athletes versus the non-RASH athletes in their HA coordination, which indicates the progression from hip flexion to extension as the athlete approaches toe off. There is a noticeable increase in the HA coordination angle during the second half stance phase of running in the RASH athletes compared to the non-RASH athletes. In the pre-test analysis, the RASH players are seen to have a 212.7° HA angle representing a late in-phase hip dominant motion. The training led to an increase in the post-training HA coordination angle at 235.5°, which indicates a transition into an in-phase ankle-dominant movement [28]. Non-RASH coupling angles were higher, approaching 270°. The medium effect size (0.075) suggests a meaningful finding that the RASH group appears to possess sensorimotor deficits at the start of camp in this subphase, but underwent reductions in sensorimotor deficits after training (partial recovery); specifically, the ankle contribution increased but was still less than non-RASH group. It is plausible that more time (>6 weeks) may be needed to restore ankle control at push-off. Coordination variability was greatest in the second half of stance when compared to all subphases for both groups (63.8-67.1°). 

First Half Swing

During the first half of the swing subphase, coordination angles for both groups shift back to an in-phase hip dominant pattern (16.9-19.1°), reflecting coordinated joint flexion as the limb advanced into non-weight-bearing. Of all gait cycle subphases, coordination variability was lowest for the first half of swing, suggesting that less adaptability is required in this subphase. Both coordination angle and coordination variability in RASH athletes were lower than in non-RASH athletes, and both increased post-training. The medium effect size of 0.121 for coordination variability suggested a potential influence of training and recent ankle sprain history on variability.

Second Half Swing

During the second half of the swing, RASH athletes continued to show more in-phase hip dominance (184.1-187.3°), while the non-RASH athletes transitioned from an anti-phase hip dominance to in-phase hip dominance coordination following training (174.9-184.1°). However, the effect size was small (0.011). In contrast to the non-RASH group, variability decreased for the RASH group. In this subphase, the leg is preparing for initial contact, and RASH athletes may have compensated to attenuate the foot contact with the ground. This adaptation could potentially be negative since too large a variability indicates a rigid pattern with increased injury risk [14-16].

Knee-ankle coordination

The angle-angle plots show that the RASH athletes had limited ankle dorsiflexion at the pre-test. Limited ankle dorsiflexion, especially during stance subphases, is common after ankle sprains [9]. After training camp, RASH athletes show increased ankle dorsiflexion paired with an increase in knee flexion during the swing phase, with increased ankle dorsiflexion in the later stance phase. The improvements in ankle motion to the HA and KA couplings are noteworthy.

First Half Stance

There is a noticeable difference in the effects of the NFL draft preparation training on the first phase of the running gait between RASH and non-RASH athletes. In athletes with recent ankle sprain history, the training led to a decrease in KA coordination angle from 97° to 85.5°, while the non-injured athletes saw a slight increase in the first-half stance phase KA coordination angle from 93.6° to 95.6°. Although there is a negligible change in the coordination pattern classification in the non-injured athletes, the decrease in the RASH group shifts them from an ankle-dominant anti-phase to an ankle-dominant in-phase movement. This shift to an in-phase ankle-dominant movement at this point may indicate strengthening of the ankle joint in these athletes following their training via altered load attenuation strategies or neuromuscular adaptations secondary to the recent ankle sprain (medium effect size: 0.093). Variability was highest for this phase for both groups and remained stable with training (68.5-69.2°). A flexible neuromuscular strategy regarding KA coupling appears advantageous for initial contact and weight acceptance [7].

Second Half Stance

In the second half of stance, both groups transitioned to more anti-phase knee-dominant coordination, with RASH maintaining slightly greater knee dominance. This greater reliance on proximal control may be a compensatory strategy for push-off, but the effect size was very small at 0.009. Variability was similar between the two groups and remained stable at approximately 50°.

First Half Swing

During the first half of swing, both groups showed predominantly in-phase knee-dominant coordination supporting coordinated joint flexion. There is a marginal decrease in coordination angles following training in the RASH group, while the non-RASH group exhibits a slight increase in the same measurements. Variability in RASH athletes were noticeably decreased following training camp compared to that of non-RASH athletes. The RASH group developed a 2.2° decrease in coordination variability compared to the 0.3° decrease in the non-RASH group. The effect size (0.058) implies a potential interaction, and perhaps higher variability in the first half of swing for KA is not beneficial.

Second Half Swing

During the second half of swing, coordination angles shift toward greater in-phase knee dominance, and variability decreases in preparation for initial contact. RASH did have small reductions in the coordination angle (4.4°) that produced a medium effect size (0.091), suggesting residual motor control deficits that may affect the pre-activation of the ankle musculature for weight-bearing. The pattern did stay in-phase proximal dominant. Additionally, the athletes in the non-RASH cohort began with a lower baseline for their coordination variability in the second-half swing phase at 37.3° with a slight increase, while the RASH group began at a higher baseline of 45.5° with a similar marginal increase, small to medium effect size at 0.05. Increases in variability within the later swing phases are consistent as a form of motor control adaptation for those with ankle injury history [10].

In American football, ankle sprains are no stranger. With nearly 53% of NFL Combine athletes sustaining an ankle injury in their athletic career and 14% of NFL injuries being ankle injuries, it is important to educate athletes, coaches, and trainers on appropriate measures to return to a full range of motion and strength in supporting muscles and supporting joints. With approximately 90% of athletes who suffer a high ankle returning to play within 80-200 days [32], it is important for sports performance and medicine practitioners to best prepare the football athletes to prevent further injuries. Despite multiple protocols and medical interventions available to these athletes, ankle injuries still negatively affect performance even when injuries occur years prior [32]. Kwon et al. argue that the importance of a stable joint-coupling relationship and controlled coordination variability is reducing persistent ankle damage [20]. By strengthening and improving mobility of the ankle joint to improve dorsiflexion in the stance phase of gait through rotational stretches and focused exercises, there is a greater ability to prevent future injuries in football athletes. Our findings are consistent with previous studies claiming greater dorsiflexion in the middle and late phases of the running gait in athletes recovering from ankle sprain [33]. With proper mobility and strength training in recovering athletes, there is an increased range of motion in these joints that support the dynamic balance and stability in the running gait for these elite athletes.

While this study presents promising findings for restoring lower limb function, it is not without limitations. We did not assess for chronic ankle instability, in which 40%-70% of individuals who sustain an ankle sprain will develop [34]. Our study limited the history of ankle sprains to six months prior to the initial data collection period, and we did not have diagnostic imaging to confirm any residual abnormalities. We suggest this to future researchers so anatomical limitations may be considered in concert with neuromuscular ones. We also did not ensure subjects wore the same footwear for both testing sessions, which may have affected subtle running biomechanics. We were also unable to obtain clinical measures, such as strength and range of motion, that would have enriched the interpretation of these findings. Finally, the training camp in this study employed interventions designed to fine-tune strength, power, and agility in accordance with specific criteria used during an NFL tryout (e.g., vertical jump, 36.6-m dash, and 18.3-m shuttle run). While our results demonstrate that even elite athletes have deficits that can be modified, the generalizability of the results to the general population or other fitness participants and sports may be limited.

Future directions may include assessing intersegmental coordination of players with other common injuries, such as hamstring strains. Evaluating in the off-season may offer useful clinical and scientific insights into the influence of targeted rehabilitation programs on coordination.

## Conclusions

The authors report a pilot study analyzing intersegmental coordination and coordination variability of hip, knee, and ankle joints following a recent ankle sprain history in elite American football athletes preparing for the NFL draft. These athletes completed an intensive and focused six-week training program, allowing a comparison of the hip-ankle coordination and knee-ankle coordination before training and after training. Although we did not find any significant differences in the hip-ankle and knee-ankle coordination angles or the coordination variability, we did find noteworthy results that highlight the persistence of movement pattern deficits even in medically cleared elite performers, as well as potential training effects on the patterns. First, the RASH group presented with altered HA coupling patterns that reflected a greater reliance on proximal control, particularly during propulsion in the second half of stance. Ankle dorsiflexion, particularly, was enhanced at midstance, as was hip extension at take-off. Second, the KA coordination patterns seemed less affected by ankle sprain history, as RASH athletes exhibited the same dominance and phase categorization as non-RASH athletes. Training appeared to benefit the RASH group by producing greater ankle dorsiflexion, accompanied by increased knee flexion during the swing phase of the running gait cycle, with increased dorsiflexion during the second half of the stance phase. Third, HA and KA variability did not appear as affected by recent ankle sprain history or training. Variability was lowest for the HA first half of swing subphase, and minor increases, as shown in the current study, may be important for similar cohorts. Our findings suggest that recent (≤6 months) but asymptomatic ankle sprain history impacts not only ankle joint motion but also coordinative movement with the knee and hip. Further, with a focused approach and training plan, these elite-level athletes can develop their neuromuscular connection to enhance intersegmental coordination, possibly impacting sport performance and injury risk.

## References

[1] Chandran A, Moffit RE, DeJong Lempke AF (2023). Epidemiology of lateral ligament complex tears of the ankle in National Collegiate Athletic Association (NCAA) Sports: 2014-15 through 2018-19. Am J Sports Med.

[2] Roos KG, Kerr ZY, Mauntel TC, Djoko A, Dompier TP, Wikstrom EA (2017). The epidemiology of lateral ligament complex ankle sprains in National Collegiate Athletic Association Sports. Am J Sports Med.

[3] Hootman JM, Dick R, Agel J (2007). Epidemiology of collegiate injuries for 15 sports: summary and recommendations for injury prevention initiatives. J Athl Train.

[4] Chandran A, Morris SN, Powell JR, Boltz AJ, Robison HJ, Collins CL (2021). Epidemiology of injuries in National Collegiate Athletic Association men’s football: 2014-2015 through 2018-2019. J Athl Train.

[5] Mulcahey MK, Bernhardson AS, Murphy CP (2018). The epidemiology of ankle injuries identified at the National Football League combine, 2009-2015. Orthop J Sports Med.

[6] Wikstrom EA, Cain MS, Chandran A, Song K, Regan T, Migel K, Kerr ZY (2021). Lateral ankle sprain and subsequent ankle sprain risk: a systematic review. J Athl Train.

[7] Alghadir AH, Iqbal ZA, Iqbal A, Ahmed H, Ramteke SU (2020). Effect of chronic ankle sprain on pain, range of motion, proprioception, and balance among athletes. Int J Environ Res Public Health.

[8] Halabchi F, Hassabi M (2020). Acute ankle sprain in athletes: clinical aspects and algorithmic approach. World J Orthop.

[9] Drewes LK, McKeon PO, Paolini G, Riley P, Kerrigan DC, Ingersoll CD, Hertel J (2009). Altered ankle kinematics and shank-rear-foot coupling in those with chronic ankle instability. J Sport Rehabil.

[10] Wanner P, Schmautz T, Kluge F, Eskofier B, Pfeifer K, Steib S (2019). Ankle angle variability during running in athletes with chronic ankle instability and copers. Gait Posture.

[11] Sterett WI, Briggs KK, Farley T, Steadman JR (2006). Effect of functional bracing on knee injury in skiers with anterior cruciate ligament reconstruction: a prospective cohort study. Am J Sports Med.

[12] Reljic D, Hässler E, Jost J, Friedmann-Bette B (2013). Rapid weight loss and the body fluid balance and hemoglobin mass of elite amateur boxers. J Athl Train.

[13] Varma V, Trkov M (2024). Investigation of intersegmental coordination patterns in human walking. Gait Posture.

[14] Stergiou N, Harbourne R, Cavanaugh J (2006). Optimal movement variability: a new theoretical perspective for neurologic physical therapy. J Neurol Phys Ther.

[15] Davis K, Williams JL, Sanford BA, Zucker-Levin A (2019). Assessing lower extremity coordination and coordination variability in individuals with anterior cruciate ligament reconstruction during walking. Gait Posture.

[16] Hewett TE, Myer GD, Ford KR (2005). Biomechanical measures of neuromuscular control and valgus loading of the knee predict anterior cruciate ligament injury risk in female athletes: a prospective study. Am J Sports Med.

[17] Sheikhhoseini R, Abdollahi S, Salsali M, Anbarian M (2025). Biomechanical coordination and variability alters following repetitive movement fatigue in overhead athletes with painful shoulder. Sci Rep.

[18] Yu P, Cen X, Mei Q, Wang A, Gu Y, Fernandez J (2024). Differences in intra-foot movement strategies during locomotive tasks among chronic ankle instability, copers and healthy individuals. J Biomech.

[19] Chang R, Van Emmerik R, Hamill J (2008). Quantifying rearfoot-forefoot coordination in human walking. J Biomech.

[20] Kwon YU, Harrison K, Kweon SJ, Williams DS 3rd (2020). Ankle coordination in chronic ankle instability, coper, and control groups in running. Med Sci Sports Exerc.

[21] Needham RA, Naemi R, Hamill J, Chockalingam N (2020). Analysing patterns of coordination and patterns of control using novel data visualisation techniques in vector coding. Foot (Edinb).

[22] (2013). World Medical Association Declaration of Helsinki: ethical principles for medical research involving human subjects. JAMA.

[23] Hafer JF, Boyer KA (2017). Variability of segment coordination using a vector coding technique: reliability analysis for treadmill walking and running. Gait Posture.

[24] Winter DA (2009). Biomechanics and Motor Control of Human Movement. Wiley & Sons.

[25] Dixon PC, Loh JJ, Michaud-Paquette Y, Pearsall DJ (2017). biomechZoo: an open-source toolbox for the processing, analysis, and visualization of biomechanical movement data. Comput Methods Programs Biomed.

[26] Novacheck TF (1998). The biomechanics of running. Gait Posture.

[27] Hamill J, Haddad JM, McDermott WJ (2000). Issues in quantifying variability from a dynamical systems perspective. J Appl Biomech.

[28] Needham RA, Naemi R, Chockalingam N (2015). A new coordination pattern classification to assess gait kinematics when utilising a modified vector coding technique. J Biomech.

[29] Cohen J (2013). Statistical Power Analysis for the Behavioral Sciences.

[30] Steiger JH (2004). Beyond the F test: effect size confidence intervals and tests of close fit in the analysis of variance and contrast analysis. Psychol Methods.

[31] Hafer JF, Peacock J, Zernicke RF, Agresta CE (2019). Segment coordination variability differs by years of running experience. Med Sci Sports Exerc.

[32] Desai SS, Dent CS, Hodgens BH (2022). Epidemiology and outcomes of ankle injuries in the National Football League. Orthop J Sports Med.

[33] Yang X, Jiang H, Yu P, Mei Q, Fernandez J, Gu Y (2023). Analysis of segmental coordination in the lower extremity using vector coding: a pilot return-to-play study of acute ankle sprain. Acta Bioeng Biomech.

[34] Gribble PA, Bleakley CM, Caulfield BM (2016). Evidence review for the 2016 International Ankle Consortium consensus statement on the prevalence, impact and long-term consequences of lateral ankle sprains. Br J Sports Med.

